# Deep learning-based automatic segmentation for size and volumetric measurement of breast cancer on magnetic resonance imaging

**DOI:** 10.3389/fonc.2022.984626

**Published:** 2022-08-11

**Authors:** Wenyi Yue, Hongtao Zhang, Juan Zhou, Guang Li, Zhe Tang, Zeyu Sun, Jianming Cai, Ning Tian, Shen Gao, Jinghui Dong, Yuan Liu, Xu Bai, Fugeng Sheng

**Affiliations:** ^1^ Department of Radiology, The Fifth Medical Center of Chinese PLA General Hospital, Beijing, China; ^2^ Chinese PLA General Medical School, Beijing, China; ^3^ Keya Medical Technology Co., Ltd., Beijing, China

**Keywords:** deep learning, breast cancer, magnetic resonance imaging, volumetric measurement, automatic segmentation

## Abstract

**Purpose:**

In clinical work, accurately measuring the volume and the size of breast cancer is significant to develop a treatment plan. However, it is time-consuming, and inter- and intra-observer variations among radiologists exist. The purpose of this study was to assess the performance of a Res-UNet convolutional neural network based on automatic segmentation for size and volumetric measurement of mass enhancement breast cancer on magnetic resonance imaging (MRI).

**Materials and methods:**

A total of 1,000 female breast cancer patients who underwent preoperative 1.5-T dynamic contrast-enhanced MRI prior to treatment were selected from January 2015 to October 2021 and randomly divided into a training cohort (*n* = 800) and a testing cohort (*n* = 200). Compared with the masks named ground truth delineated manually by radiologists, the model performance on segmentation was evaluated with dice similarity coefficient (DSC) and intraclass correlation coefficient (ICC). The performance of tumor (T) stage classification was evaluated with accuracy, sensitivity, and specificity.

**Results:**

In the test cohort, the DSC of automatic segmentation reached 0.89. Excellent concordance (ICC > 0.95) of the maximal and minimal diameter and good concordance (ICC > 0.80) of volumetric measurement were shown between the model and the radiologists. The trained model took approximately 10–15 s to provide automatic segmentation and classified the T stage with an overall accuracy of 0.93, sensitivity of 0.94, 0.94, and 0.75, and specificity of 0.95, 0.92, and 0.99, respectively, in T1, T2, and T3.

**Conclusions:**

Our model demonstrated good performance and reliability for automatic segmentation for size and volumetric measurement of breast cancer, which can be time-saving and effective in clinical decision-making.

## Introduction

Breast cancer is one of the most common malignancies afflicting women worldwide (1). Tumor size has been thought as an indispensable prognostic factor. An accurate preoperative measurement of breast cancer size is essential for surgical resection and the formulation of a chemotherapy regimen (2–4). Furthermore, monitoring the change of tumor volume during treatment is an important reference for response evaluation criteria in solid tumors (5). Thus, it is crucial to measure size and volume accurately in the clinical course.

Medical imaging, which is superior in measuring tumor size and volume, might be used to obtain anatomic information accurately and non-invasively (6–10). Among the imaging methods, magnetic resonance imaging (MRI) is a better diagnostic technique with the highest resolution and quantitative information for preoperative prediction and prognosis evaluation (11–14). However, it takes considerable time and a great deal of expertise to process images by trained radiologists. In addition, due to differences in diagnostic skills, there are inter- and intra-observer variations among radiologists and problems with decision fatigue (15, 16).

Artificial intelligence (AI) aiding medical imaging technologies exceeded the detection capabilities of radiologists in some applications, complemented clinical decision-making, and streamlined preoperative image evaluation. Automated processing by AI computational tools is a more efficient detection approach to measure the volume and the size of a tumor within a reasonable amount of time. It has great reference significance for guiding the clinical development of follow-up treatment plans and avoiding inaccurate measurement incurred by some inexperienced radiologists (17). In addition, some studies indicated that the presence of tumor necrosis correlated with tumor grade, aggressiveness, unfavorable long-term outcomes, and improved response to neoadjuvant chemotherapy (18–20). Measuring the necrosis and the cystic components manually is labor-consuming, but using AI technology improves the efficiency and provides more intuitive parameters for radiologists.

Segmentation plays a significant role in image analysis, including detection, feature extraction, classification, and treatment (21, 22). Automatic and semiautomatic segmentation can alleviate the labor-intensive problems and eliminate the high variability between intra- and inter-observers (23). Moreover, deep learning, as a subset of AI, is a promising method to make a tremendous progress in automatic segmentation by which more reproducible and effective texture features in different fields of image analysis are extracted (24–26). The convolutional neural network (CNN) is a sophisticated deep learning architecture, and it has been successfully applied in various areas of knowledge for digital image segmentation. The U-Net network is a fully CNN with high-performance in graphics processing unit (GPU) computing, requiring fewer training sets, and has higher segmentation accuracy compared with other CNNs (27). Among the U-Net network, Res-UNet is a semantic segmentation model which integrates residual module and U-Net network capable of effectively overcoming excessive parameters and gradient dispersion caused by the deepened network layer (28).

In this study, we developed a deep learning automatic segmentation model based on Res-UNet of preoperative MRI for breast cancer patients and assessed its reliability for size and volumetric measurement. To our knowledge, no reported research has applied deep learning to automatically segment breast cancer and quantify the volume as well as the size on MRI.

## Materials and methods

The institutional review board approved this retrospective study and waived the need for written informed consent.

### Study design

**Figure 1:**
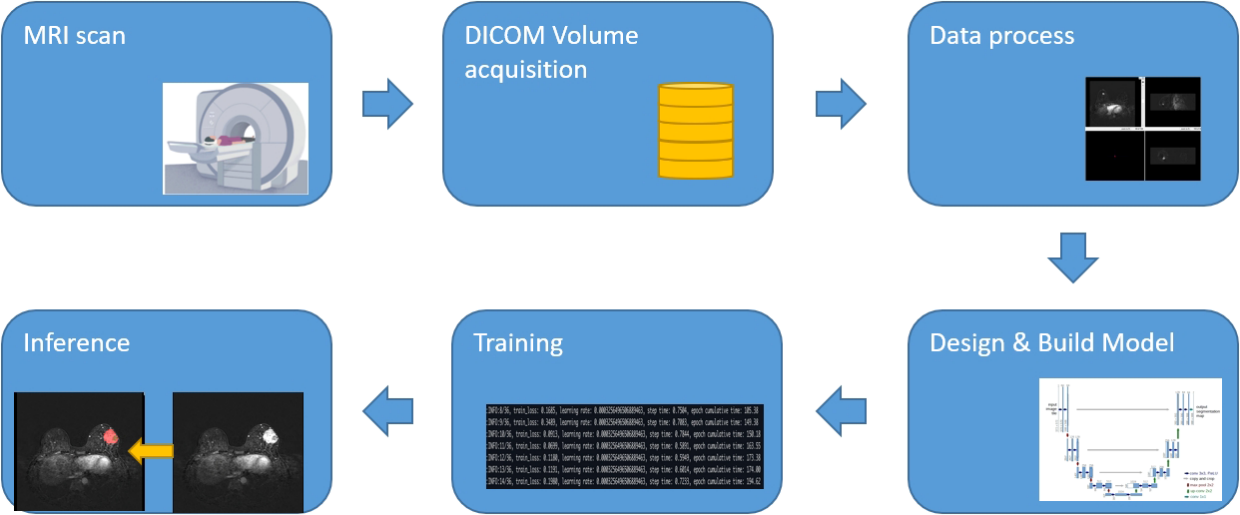
Workflow diagram.

The workflow of the process is illustrated in **Figure 1**, including the following three steps: (1) acquisition of MRI, data annotation, automatic segmentation, image pre-processing, augmentation, and post-processing, (2) designing and building the algorithm, and (3) training and inference.

### Patient selection and data annotation

All selected female patients were diagnosed with breast cancer who underwent preoperative breast MRI prior to treatment from January 2015 to October 2021. The inclusion criteria were as follows: (1) diagnosed with breast carcinoma pathologically, (2) underwent MRI prior to treatment, (3) with complete clinical and pathological data, and (4) whose digital imaging and communications in medicine pixel data had no corruption and which were scanned under the same MR protocol. The exclusion criteria were as follows: (1) received any therapy before MRI and (2) non-mass enhancement breast cancer or normal in MRI.

According to the Cancer Staging Manual of the American Joint Committee on Cancer, the system clarified that the tumor (T) stage is based on the size of the invasive components of the longest tumor dimension (in the setting of multiple masses). Our study classified the tumor into three T categories: the size of T1 is not greater than or equal to 20 mm, the size of T2 is larger than 20 mm and not greater than 50 mm, and the size of T3 is equal or greater than 50 mm. A total of 1,000 patients were randomly divided into group 1 (*n* = 230), group 2 (*n* = 720), and group 3 (*n* = 50). The following ratios were used: 80% training cohort and 20% testing cohort to balance the test samples. Thus, we selected 45, 143, and 12 cases relatively for three T categories as testing cohort. In addition, 31 cases with cystic or necrotic changes were enrolled in our study.

### MRI acquisition

All patients were scanned using a 1.5-T system (Magnetom Espree Pink; Siemens, Erlangen, Germany), which is equipped with an eight-channel phased-array surface coil for the breast. The patients were examined in the prone position with both breasts positioned in the coil cavity. Axial T1WI [repetition time/echo time (TR/TE), 8.7/4.7 ms; slice thickness, 1.1 mm]. Dynamic contrast-enhanced MRI (DCE-MRI) used a fast, small-angle excitation, three-dimensional imaging (3D-FLASH) sequence and fat-saturated axial T1WI: TR/TE, 4.53/1.66 ms; slice thickness, 1.1 mm. Before the contrast agent was injected, it needed to be scanned one time. After that, the contrast agent, gadopentetate dimeglumine, was injected with a high-pressure syringe at a speed of 2.0 ml/s, and then 30 ml normal saline was injected at the same speed to flush the catheter. Images of each phase were subtracted automatically at the same time.

### Delineation of ROIs by iterative workflow

**Figure 2:**
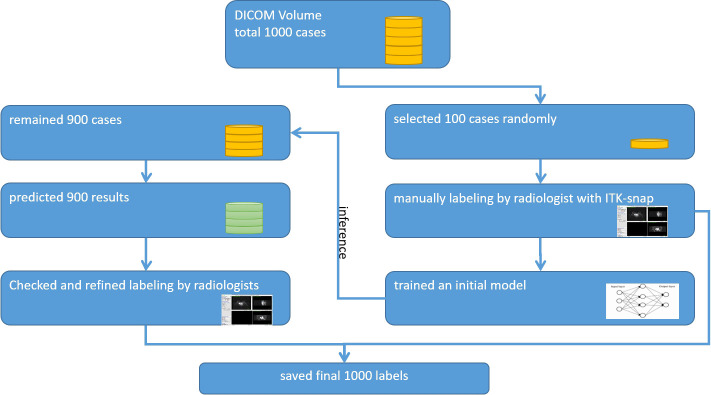
Iterative labeling workflow.

A radiologist used the ITK-SNAP software (www.itksnap.org) to review the first DCE-MRI subtraction images, this being the most critical and the clearest phase of breast cancer evaluation for further analysis. An iterative-label workflow was used to delineate the regions of interest (ROIs) in the early stage to get the ideal labels. It included an initial network model which was trained on our in-house dataset from 100 patients’ ROIs and the pre-trained model which was applied to the remaining patients’ ROIs and achieved coarse labels. After that, two radiologists checked and refined the manual revision. The iterative workflow is shown in **Figure 2**.

### Image processing

We designed a fully automatic CNN-based segmentation network and built an end-to-end workflow based on Medical Open Network for AI^1^ platform, including pre-processing, data loader, augmentation, network building, post-processing, and quantification.

### Pre-processing

Three steps including intensity normalization, respacing, and crop patches were performed before the training model. *Z*-score normalization was suitable for variable intensity ranges. We calculated the intensity ranges, clamped the voxels from 0.5 to 99.5%, calculated the mean and standard deviation (SD) of each case, and used the equation to normalize the images. It is defined as shown in Equation (1):


 (1) $$\text{images}=\frac{\text{images }-\text{ mean}}{\text{SD}}$$

To automatically adapt to any new dataset, we calculated the mean spacing of all training cohorts to define the standard target. Any data needed to be resized to the target before training and inference. Most cases had the dimension of the width and the height as 384 and the depth from 128 to 320. Due to the limitation of the GPU memory size, it was challenging to send the whole image to the network. In this situation, we first calculated the average area of the lesions and set a minimum cropping patch size which can include the central regions. The cropping patch size must also be a multiple of 2 to be suitable for most regular models. According to the statistics, a patch with a 96*96*96 size was the best choice for our algorithm.

To ensure the balance of positive and negative samples for network training, we randomly selected the cropping patches with the center point at the foreground or background area with half-to-half probability. According to our experiments, crop patches with the likelihood of 2:1 between positive and negative areas can also get a similar performance.

### Augmentation

**Figure 3:**
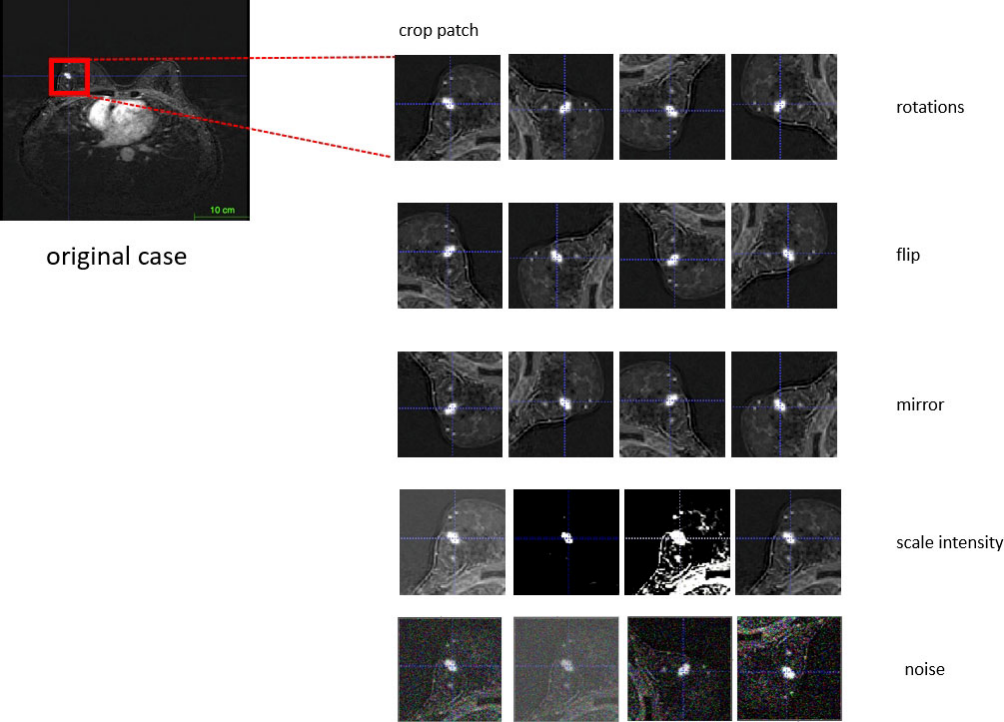
Workflow of data augmentation.

Our algorithm implemented data augmentation to make the model more robust during training steps: random zoom, random scale intensity value, random shift intensity range, random Gaussian noise, random crop fore/background, random rotation with 90°, and random elastic transformation (**Figure 3**).

### Res-UNet network building

**Figure 4:**
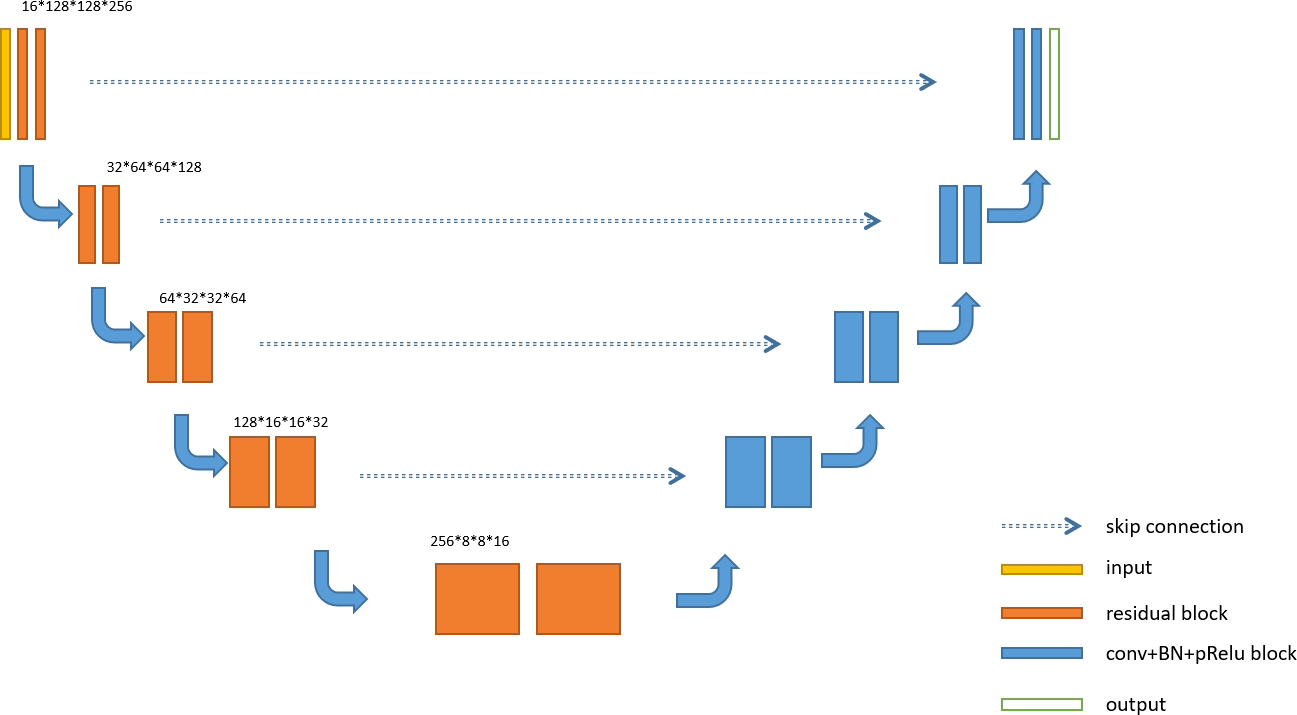
Res-UNet architecture.

**Figure 5:**
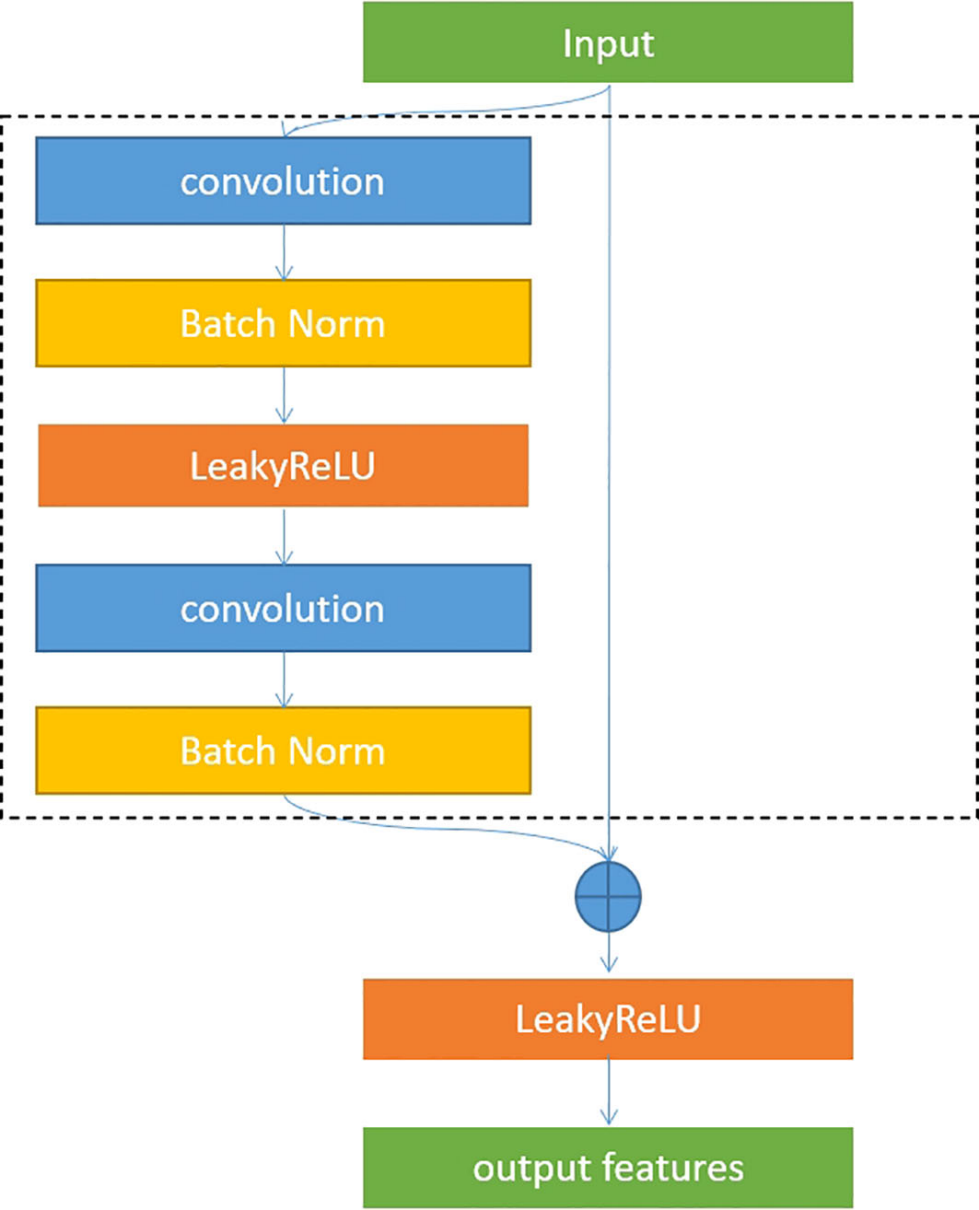
Residual blocks.

U-Net is an overall architecture for 2D and 3D images in medical image processing. Our study designed a robust U-Net-based network called Res-UNet with the residual blocks in the encoder part. **Figure 4** shows the architecture of our designed model. In the encoder part, we used residual blocks to extract features. Skip connection was a classical operation from U-Net and might focus on the extracted features from different layer levels. It was well suited for medical images since lesion targets from different scale levels included different features. **Figure 5** illustrates the residual blocks. The solid line carrying the layer input to the addition operator was a residual connection. The residual connection might effectively avoid gradient disappearance, especially in deeper layers. We combined the residual connection blocks with the U-Net skip connection to design an efficient network, which might help us obtain the accurate prediction results of lesion segmentation.

### Optimizer and loss function

In our algorithm, we used Adam optimizer, dice similarity coefficient (DSC), and cross-entropy loss. The equations are shown in Equations (2)–(4):


(2) loss=DSC_loss * 0.5+ cross_entropy_loss * 0.5

(3) $$\text{DSC}\_\text{loss}=1\;-\;\frac{2*\left|\text{X}\cap^{}\text{Y}\right|}{\left|\text{X}\right|\;+\;\left|\text{Y}\right|}$$

(4) $$\text{cross}\_\text{entropy}\_\text{loss}=\;-\sum_{m=0}^{N}y_{m}\ln\left(\sigma \left(x_{i}\right)\right)$$

### Post-processing

Usually, the tumor is an agglomerate region. We removed the outliers with less than 30 voxels in a connected region to avoid the noise of predicted results for the accuracy. Testing time augmentation is an effective way to improve accuracy in the inference step. We only applied rotation with 90°, 180°, and 270° to repeat the inference in one case to save time. It might improve the DSC of the testing cohort with 1 to 2%. We also tried a multi-model ensemble and trained the same Res-UNet network with different epochs. The ensemble also improved the accuracy by around 1%.

### Measurement of pixel level

**Figure 6:**
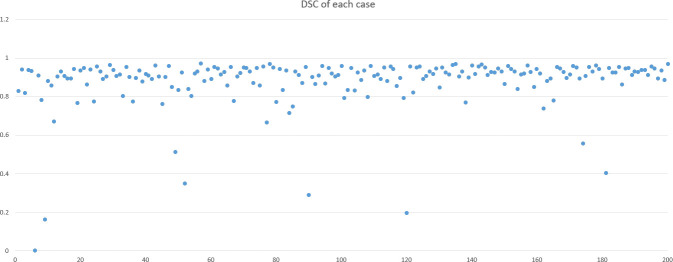
Dice similarity coefficient and intersection over union performance.

**Figure 7:**
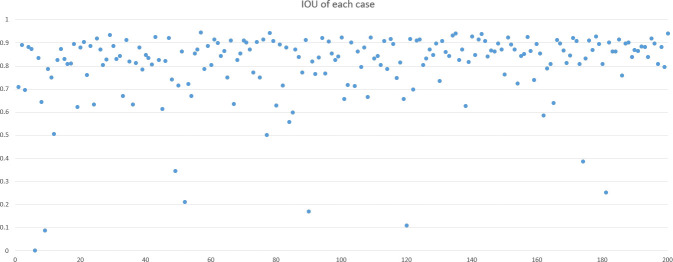
Dice similarity coefficient and intersection over union performance.


DSC and intersection over union (IOU) are commonly used metrics in segmentation algorithms. We use these two coefficients to evaluate our segmentation performance. These coefficients are spatial overlap indexes utilizing segmentation in MRI as reproducibility validation metrics. The definition of DSC and IOU are shown as Equation (5) and Equation (6). **Figures 6**, **7** show the DSC and IOU performance of our segmentation.

(5) $$\text{DSC}=\;\frac{2*\left|\text{X}\cap^{}\text{Y}\right|}{\left|\text{X}\right|\;+\;\left|\text{Y}\right|}$$

(6) $$\text{IOU}=\;\frac{\left|\text{X}\cap^{}\text{Y}\right|}{\left|\text{X U Y}\right|}$$

**Figure 8:**
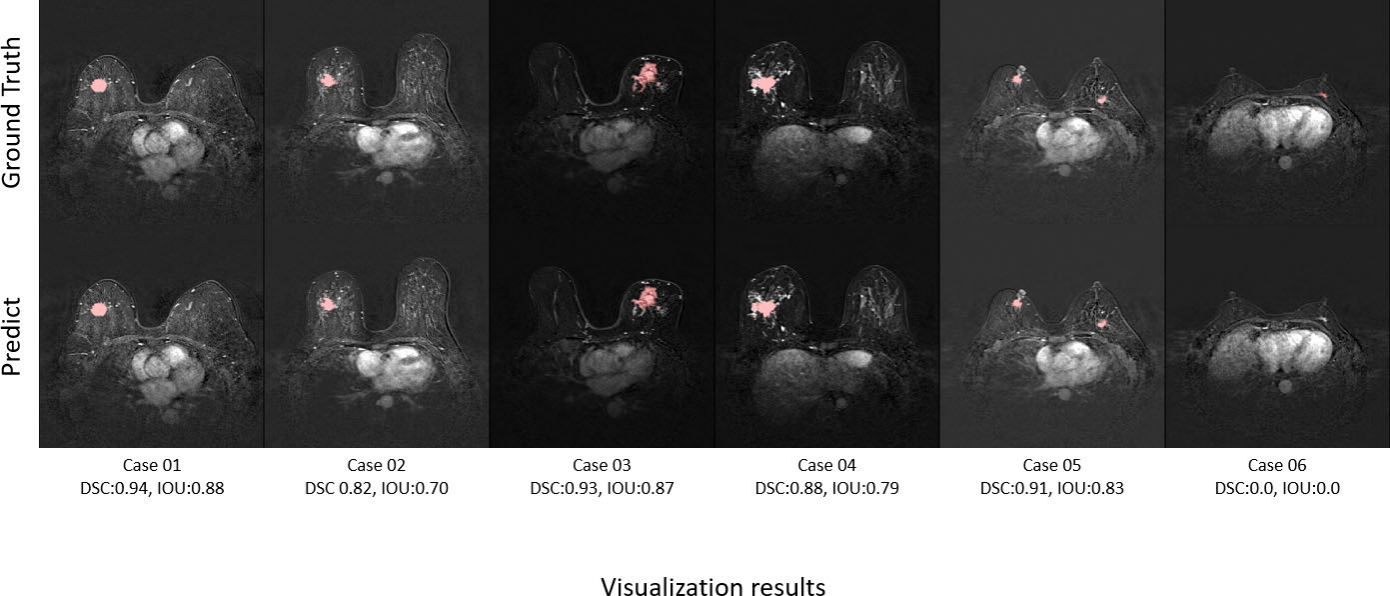
Six cases showing comparisons between the ground truth and our predicted results.

**Figure 8** shows the ground truth and our predicted results. The comparison indicated that case 01 to case 05 get the accurate results with DSC of around 0.9 and IOU of around 0.85. In case 06, a small lesion region was not segmented by the model; thus, the DSC and IOU are 0.0. We thought that the lesion was too small and quite similar to fat. This will be solved with the more various training cohorts.

### Measurement of size and volume

**Figure 9:**
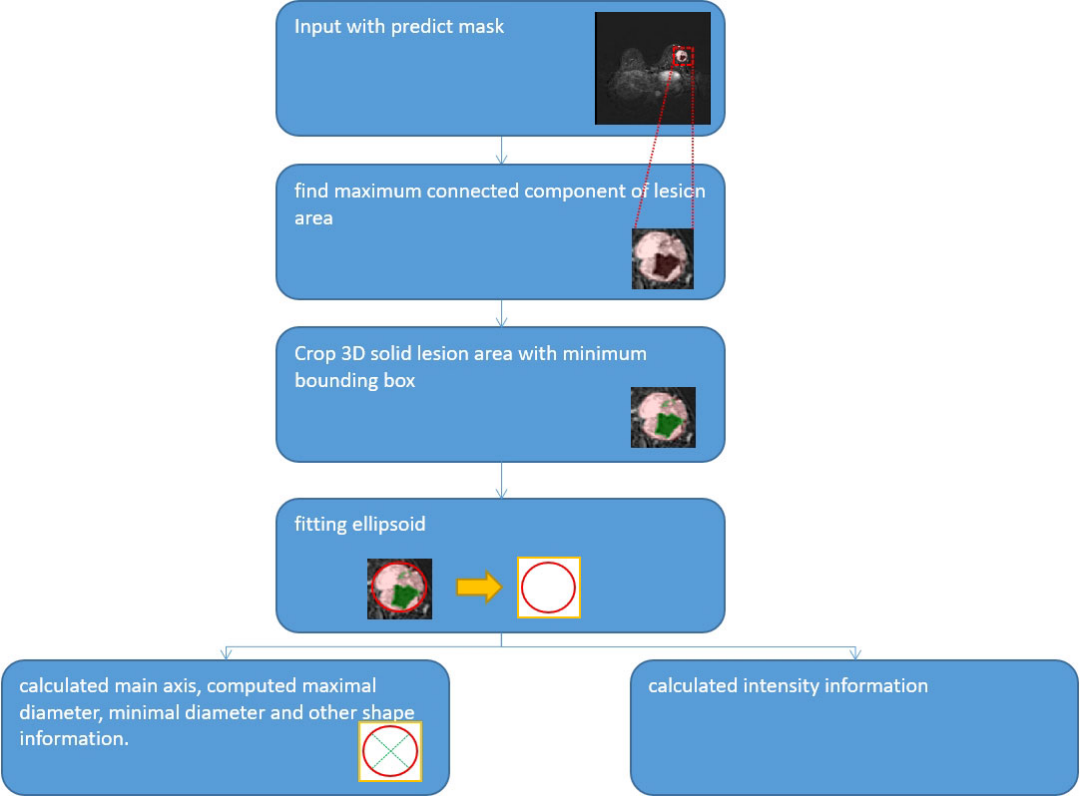
Workflow of measurement.

**Figure 10:**
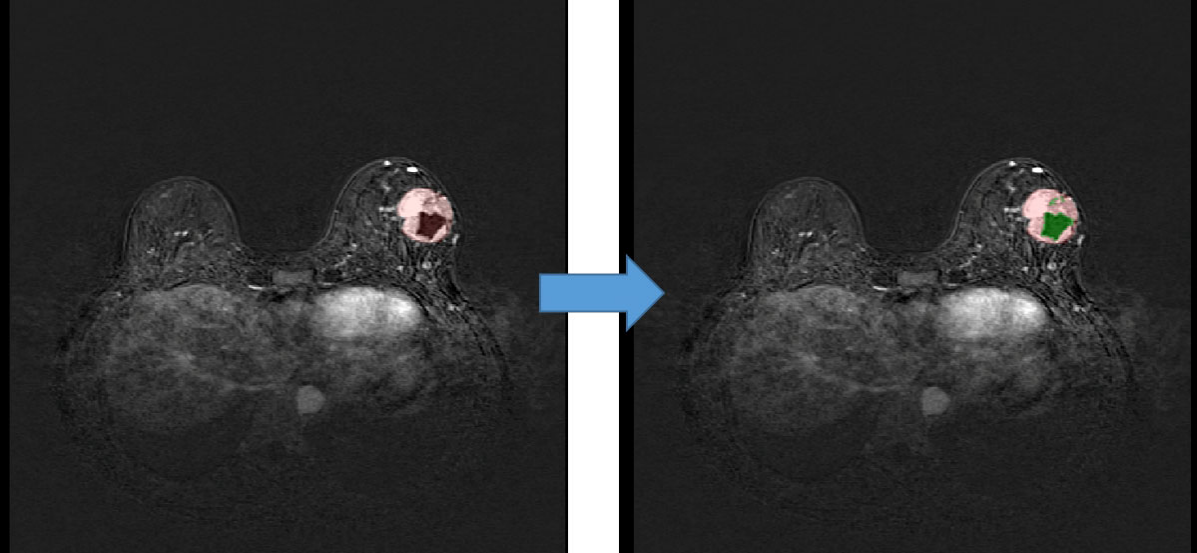
Regions of interest of the areas with cystic or necrotic changes. The green part shows the classification from the Otsu’s method.

**Figure 11:**
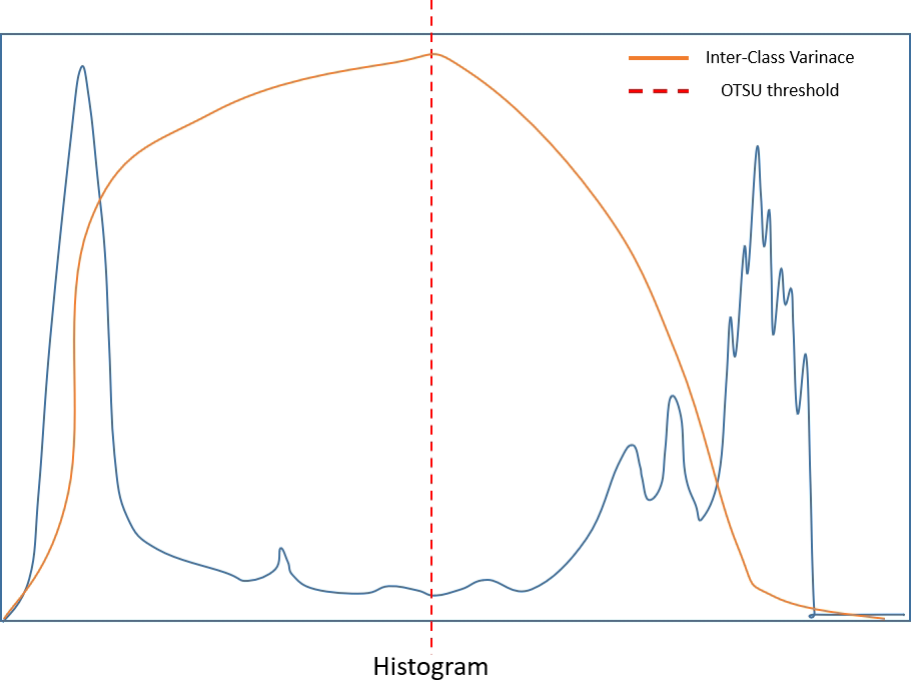
Histogram of the lesion areas.

We used quantification indexes to calculate shape-based features such as “maximum 3D diameter, 3D mesh volume, minimal diameter, maximal diameter, volume”. The method firstly extracted the largest tumor area, found the maximum connected components, cropped a 3D region with its solid components fitting as an ellipsoid, and then calculated the factors which might influence shape information. These were all based on the pyradiomics library (**Figure 9**). We also used the Otsu’s method, which is a classical intensity-based method, to divide the cystic degeneration or necrosis region manifested as hypointense in the central or paracentral area on DCE-MRI (**Figure 10**). It iteratively searched for the threshold that minimizes the within-class variance from the histogram. **Figure 11** shows the histogram of the tumor areas calculated by Otsu’s method. Moreover, the maximum value is the threshold to find the interclass variance. After that, we used the threshold as pixel intensity value to segment cystic or necrotic change areas. So far, the central part of the cystic or necrotic areas could be extracted. The outliers, a number of voxels less than 30, were removed in each connected component area.

### Statistical analysis

The automatic segmentation performance was evaluated with DSC. The method performance of classifying the size according to T stage was assessed with accuracy, sensitivity, and specificity. Intraclass correlation coefficient (ICC) was adopted to measure the agreement between the size and the volumetric parameters of the predicted results and the GT results. All statistical analyses were conducted using Python version 3.8 (www.python.org) and SPSS 25.0 software package.

## Results

**Figure 12:**
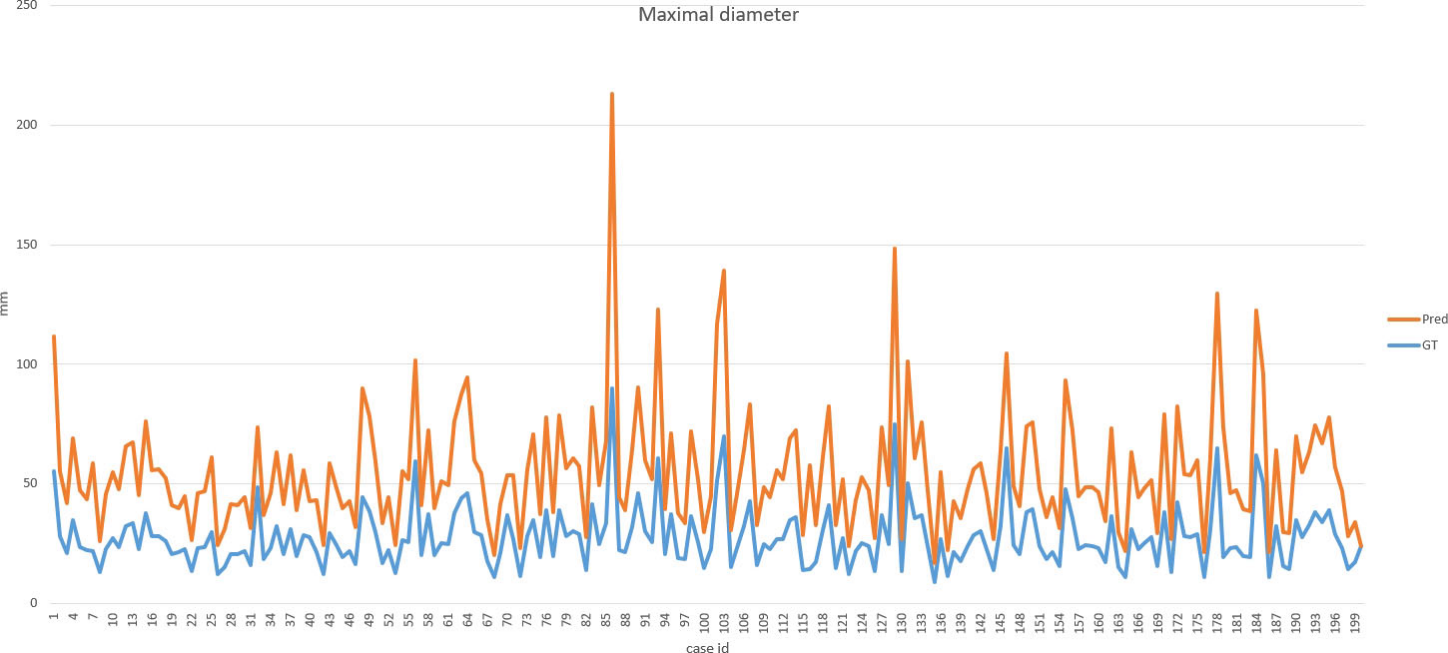
Final metrics of the predicted results.

**Figure 13:**
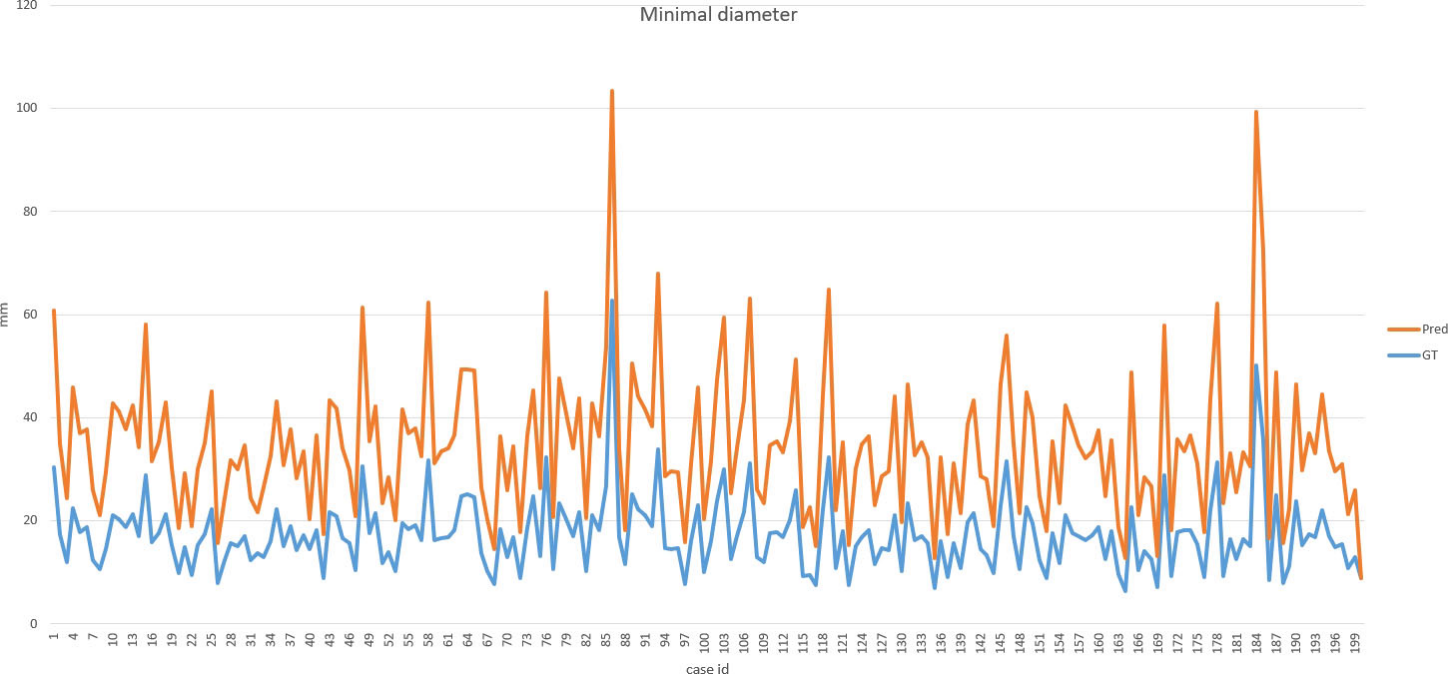
Final metrics of the predicted results.

**Figure 14:**
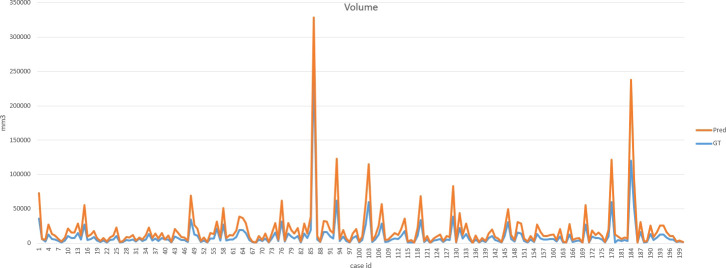
Final metrics of the predicted results.

Our improved Res-UNet got the best DSC of 0.89 among different networks. The DSCs of different networks are shown in **Table 1**. The details of DSC and IOU are presented in **Table 2**. The final metrics of the predicted outcomes in the standard-alone test cohort were accuracy = 0.93, sensitivity (T1, T2, and T3 = 0.94, 0.94, and 0.75, respectively), and specificity (T1, T2, and T3 = 0.95, 0.92, and 0.99, respectively. The detailed metrics are shown in **Tables 3**–**5**, while **Figures 12**–**14** show the details of maximal diameter, minimal diameter, and volume. **Table 6** shows the metrics of cystic or necrotic components including volume and mean intensity. A high concordance of size and volumetric parameters was shown between the deep learning segmentation-based prediction results and the GT segmentation results. For the minimal and maximal diameters, the ICC was greater than 0.95, and for volumetric measurement of mass enhancement breast cancer, the ICC was greater than 0.80 (**Table 7**). The trained model took approximately 10–15 s to provide automatic segmentation and volume analysis for each patient, while the average manual segmentation time was at least 15 min.

**Table 1:** Different networks’ dice similarity coefficient (DSC).

Metrics	UNet	nnUNet	Res-UNet
DSC	0.82	0.887	0.894
GPU memory usage in training	6 GB (batch = 8)	8 GB for normal model 32 GB for very big model	11 GB (batch = 8)

GPU, graphics processing unit.

**Table 2:** Details of dice similarity coefficient (DSC) and intersection over union (IOU).

Metrics	DSC	IOU
Average	0.88	0.80
Standard deviation	0.13	0.15

**Table 3:** Summary of geometric parameters between the prediction results and GT results.

Geometric parameters	Predict	GT
Maximum 3D diameter(mm)	33.25	34.02
3D mesh volume (mm^3^)	9,335.38	10,370.29
Minimal diameter (mm)	21.17	21.57
Maximal diameter (mm)	27.41	27.77
Volume (mm^3^)	9333.08	10416.14

GT, ground truth.

**Table 4:** Final predicted metrics of the classification.

Classification	Precision	Recall	F1-score	support
Small (<20 mm)	0.85	0.94	0.90	50
Medium (20–50 mm)	0.96	0.94	0.95	138
Large (>50 mm)	0.90	0.75	0.82	12

**Table 5:** Final predicted results of the classification.

Metrics	Small	Medium	Large
Macro average	0.91	0.88	0.89
Weighted average	0.93	0.93	0.93
Accuracy	0.93		

**Table 6:** Comparison of the volume and mean intensity between cystic or necrotic components and lesions.

Quantitative parameters	Mean of lesion	Mean of cystic component	Minimum of lesion	Minimum of cystic component	Maximum of lesion	Maximum of cystic component
Volume (mm^3^)	23,858.41	7,816.06	2,625.14	12.92	253,526.11	128,501.66
Mean intensity	362.29	198.70	204.31	105.69	582.45	321.625

**Table 7:** Agreement of size and volumetric parameters between deep learning segmentation-based prediction results and GT segmentation results.

	Prediction	GT	Intraclass correlation coefficient
Volume (mm^3^)	9,333.08 ± 13,409.19	10,416.14 ± 21,928.01	0.840
Maximal diameter (mm)	27.41 ± 13.47	27.77 ± 12.55	0.952
Minimal diameter (mm)	21.17 ± 8.63	21.57 ± 9.06	0.964

GT, ground truth.

## Discussion

Our study established a deep learning model based on the Res-UNet network architecture with DSC of 0.89 for the automatic segmentation to improve recognition efficiency and productivity with the speed of 10–15 s for one patient, eliminate inter- and intra-observer variations among breast radiologists as much as possible, and reduce information overload. Our model achieved a good performance with an overall accuracy of 0.93, sensitivity of 0.94, 0.94, and 0.75, and specificity of 0.95, 0.92, and 0.99, respectively, for three T categories in classifying the size of mass enhancement breast cancer. In addition, the model corresponded well with the GT results derived manually by radiologists in terms of size and volumetric parameters. These results implied that our framework might automate certain procedures of the preoperative evaluation for breast cancer. Although the classification capability of our model is powerful and significant, future advances which will be considered through external validation in other institutions or with larger data sets will make it more persuasive for clinical application.

Preoperative breast MRI, for its highest resolution and abundant information, becomes the most promising imaging modality for different AI applications, mainly for lesion detection and classification (12, 29). Automatically detecting and classifying (limited to benign *versus* malignant) breast lesions on MRI are relatively well-established techniques (30–33). Nevertheless, measuring the volume and the size of mass enhancement breast cancer accurately has important guidance for follow-up therapeutic decisions. In previous studies, some researchers have compared the accuracy of computer-aided detection (CAD) systems and radiologists in measuring the tumor size. The results are mostly reported such that the manual measurement of MRI is better than MRI with CAD (3, 16). However, CAD systems have limited capabilities; they also enable radiologists to process large images efficiently. Therefore, using large sample image data and more intelligent deep learning models based on neural network structures to measure the maximum diameter and solid component volume of tumors can undoubtedly improve the efficiency.

We reported an excellent performance of the model in segmentation, which is in accordance with the previous studies on breast cancer segmentation (34–37). This observation can not only provide precise segmentation and quantitative assessments of breast cancer but also assist in image analysis including detection, feature extraction, classification, and treatment. In most previous studies, tumors were segmented manually, which are prone to inter- and intra-observer variabilities (34, 38, 39). Furthermore, for the 3D medical imaging process, it is difficult and time-consuming for radiologists to measure lesions manually. Automatic segmentation and semi-automatic segmentation will reduce the time as well as improve the reliability. We used automatic segmentation which produced results consistently and reproducibly. What is more, automatically extracting an entire 3D lesion with an irregular shape only takes a few minutes, and the region in the 3D dimension by manual drawing may be discontinuous or not smooth and time-consuming.

Although several prior studies used deep learning to segment breast cancer, they did not measure the volume and the size. To the best of our knowledge, this is the first deep learning study to automatically segment mass enhancement breast cancer and measure the volume and the size on MRI. Our model also analyzed the areas with cystic or necrotic changes. Tumor necrosis has been proposed as a negative prognostic factor in some studies and could be evaluated on MRI comprehensively (40, 41). Differing from prior studies of necrosis as a predictive reference in TNBC, our study aims to automatically delineate and measure the volume of cystic and necrosis areas through our algorithm so that radiologists can intuitively find the changes in tumor components, and this would help them predict the patients’ prognosis (19, 42).

Our study had several limitations. Firstly, although this is a unicentric study with a relatively large sample size, external validation datasets from multiple centers should be set up to test the rationality of the model. Secondly, we did not simultaneously count multifocal or multicentric cancers. Non-mass enhancement breast cancer should also be tried to be divided into regions. Further research is possible in the future to expand the application scope of this model for improvement. Thirdly, from the perspective of methods, the performance of our model can still be improved. Some cases still contain false-positive regions similar to lesions with hyperintensity. We think that a false-positive-remove algorithm may suppress these error regions.

## Conclusions

Utilizing a deep learning-based algorithm based on automatic segmentation to measure the volume and the size of mass enhancement breast cancer on MRI is feasible with high accuracy and reliability, thereby reducing the effort and variabilities. Further development will be added in our study for such to be implemented into future clinical practice efficiently.

## Data availability statement

The raw data supporting the conclusions of this article will be made available by the authors, without undue reservation.

## Ethics statement

Ethical review and approval was not required for the study on human participants in accordance with the local legislation and institutional requirements. Written informed consent for participation was not required for this study in accordance with the national legislation and the institutional requirements.

## Author contributions

WY: conception, design of the study, acquisition of data, analysis and interpretation of data, and drafting the article. FS: critical revision for important intellectual content. ZS, ZT, and GL: establish the automatic segmentation with size and volumetric measurement of breast cancer. HZ, JZ, NT, SG, JD, YL, XB, and JC: acquisition of data. WY and FS: final approval of the version to be submitted. All authors contributed to the article and approved the submitted version.

## Funding

This project is supported by the National Natural Science Foundation of China (no. 21575161).

## Conflict of interest

Author GL, ZT and ZS were employed by Keya Medical Technology Co., Ltd.

The remaining authors declare that the research was conducted in the absence of any commercial or financial relationships that could be construed as a potential conflict of interest.

## Publisher’s note

All claims expressed in this article are solely those of the authors and do not necessarily represent those of their affiliated organizations, or those of the publisher, the editors and the reviewers. Any product that may be evaluated in this article, or claim that may be made by its manufacturer, is not guaranteed or endorsed by the publisher.

## Footnotes

1
https://monai.io/

## References

[1] Siegel RL Miller KD Fuchs HE Jemal A. Cancer statistics. CA: Cancer J Clin (2022) 72(1):7–33. doi: 10.3322/caac.21708 35020204

[2] Pop C Stanciu-Pop C Drisis S Radermeker M Vandemerckt C Noterman D. The impact of breast MRI workup on tumor size assessment and surgical planning in patients with early breast cancer. Breast J (2018) 24(6):927–33. doi: 10.1111/tbj.13104 30076661

[3] Park JY. Evaluation of breast cancer size measurement by computer-aided diagnosis (CAD) and a radiologist on breast MRI. J Clin Med (2022) 11(5):1172. doi: 10.3390/jcm11051172 35268263 PMC8911102

[4] Foulkes WD Reis-Filho JS Narod SA. Tumor size and survival in breast cancer–a reappraisal. Nat Rev Clin Oncol (2010) 7(6):348–53. doi: 10.1038/nrclinonc.2010.39 20309006

[5] Ding J Xiao H Deng W Liu F Zhu R Ha R. Feasibility of quantitative and volumetric enhancement measurement to assess tumor response in patients with breast cancer after early neoadjuvant chemotherapy. J Int Med Res (2021) 49(3):1410669245. doi: 10.1177/0300060521991017 PMC7944542 33682494

[6] Teichgraeber DC Guirguis MS Whitman GJ. Breast cancer staging: Updates in the AJCC cancer staging manual, 8th edition, and current challenges for radiologists, from the AJR special series on cancer staging. Am J Roentgenol (2021) 217(2):278–90. doi: 10.2214/AJR.20.25223 33594908

[7] Subhas G Shah AJ Gupta A Cook J Dubay L Silapaswan S. Review of third and fourth re-excision for narrow or positive margins of invasive and intraductal carcinoma. Int Surg (2011) 96(1):18. doi: 10.9738/1340.1 21675615

[8] Marcotte-Bloch C Balu-Maestro C Chamorey E Ettore F Raoust I Flipo B. MRI For the size assessment of pure ductal carcinoma *in situ* (DCIS): A prospective study of 33 patients. Eur J Radiol (2011) 77(3):462–7. doi: 10.1016/j.ejrad.2009.09.003 19896789

[9] Mann RM Veltman J Barentsz JO Wobbes T Blickman JG Boetes C. The value of MRI compared to mammography in the assessment of tumour extent in invasive lobular carcinoma of the breast. Eur J Surg Oncol (EJSO) (2008) 34(2):135–42. doi: 10.1016/j.ejso.2007.04.020 17574805

[10] Daniel OK Lim SM Kim JH Park HS Park S Kim SI. Preoperative prediction of the size of pure ductal carcinoma *in situ* using three imaging modalities as compared to histopathological size: does magnetic resonance imaging add value? Breast Cancer Res Tr (2017) 164(2):437–44. doi: 10.1007/s10549-017-4252-2 28439735

[11] Chen H Zhou J Chen Q Deng Y. Comparison of the sensitivity of mammography, ultrasound, magnetic resonance imaging and combinations of these imaging modalities for the detection of small (≤2 cm) breast cancer. Medicine (2021) 100(26):e26531. doi: 10.1097/MD.0000000000026531 34190189 PMC8257894

[12] Meyer-Bäse A Morra L Meyer-Bäse U Pinker K. Current status and future perspectives of artificial intelligence in magnetic resonance breast imaging. Contrast Media Mol I (2020) 2020:1–18. doi: 10.1155/2020/6805710 PMC7474774 32934610

[13] Ha R Mutasa S Karcich J Gupta N Pascual Van Sant E Nemer J. Predicting breast cancer molecular subtype with MRI dataset utilizing convolutional neural network algorithm. J Digit Imaging (2019) 32(2):276–82. doi: 10.1007/s10278-019-00179-2 PMC6456631 30706213

[14] Mann RM Cho N Moy L. Breast MRI: State of the art. Radiology (2019) 292(3):520–36. doi: 10.1148/radiol.2019182947 31361209

[15] Song SE Seo BK Cho KR Woo OH Park EK Cha J. Preoperative tumor size measurement in breast cancer patients: which threshold is appropriate on computer-aided detection for breast MRI? Cancer Imaging (2020) 20(1):32. doi: 10.1186/s40644-020-00307-0 32345364 PMC7189711

[16] Song SE Seo BK Cho KR Woo OH Son GS Kim C. Computer-aided detection (CAD) system for breast MRI in assessment of local tumor extent, nodal status, and multifocality of invasive breast cancers: preliminary study. Cancer Imaging (2015) 15(1):1. doi: 10.1186/s40644-015-0036-2 25888983 PMC4344797

[17] Meeuwis C van de Ven SM Stapper G Fernandez Gallardo AM van den Bosch MAAJ Mali WPTM. Computer-aided detection (CAD) for breast MRI: evaluation of efficacy at 3.0 T. Eur Radiol (2010) 20(3):522–8. doi: 10.1007/s00330-009-1573-5 PMC2822230 19727750

[18] Ryu DW Jung MJ Choi WS Lee CH. Clinical significance of morphologic characteristics in triple negative breast cancer. J Korean Surg Soc (2011) 80(5):301. doi: 10.4174/jkss.2011.80.5.301 22066052 PMC3204700

[19] Abdelhafez AH Musall BC Adrada BE Hess K Son JB Hwang K. Tumor necrosis by pretreatment breast MRI: association with neoadjuvant systemic therapy (NAST) response in triple-negative breast cancer (TNBC). Breast Cancer Res Tr (2021) 185(1):1–12. doi: 10.1007/s10549-020-05917-7 PMC8294182 32920733

[20] Masood S. Neoadjuvant chemotherapy in breast cancers. Women's Health (2016) 12(5):480–91. doi: 10.1177/1745505716677139 PMC5373271 27885165

[21] Siu AL U.S. Preventive Services Task Force. Screening for breast cancer: U.S. preventive services task force recommendation statement. Ann Intern Med (2016) 164(4):279–96. doi: 10.7326/M15-2886 26757170

[22] Chan H Samala RK Hadjiiski LM. CAD And AI for breast cancer–recent development and challenges. Br J Radiol (2020) 93(1108):20190580. doi: 10.1259/bjr.20190580 31742424 PMC7362917

[23] Michael E Ma H Li H Kulwa F Li J. Breast cancer segmentation methods: Current status and future potentials. BioMed Res Int (2021) 2021:9962109. doi: 10.1155/2021/9962109 34337066 PMC8321730

[24] Kickingereder P Isensee F Tursunova I Petersen J Neuberger U Bonekamp D. Automated quantitative tumour response assessment of MRI in neuro-oncology with artificial neural networks: a multicentre, retrospective study. Lancet Oncol (2019) 20(5):728–40. doi: 10.1016/S1470-2045(19)30098-1 30952559

[25] Brugnara G Isensee F Neuberger U Bonekamp D Petersen J Diem R. Automated volumetric assessment with artificial neural networks might enable a more accurate assessment of disease burden in patients with multiple sclerosis. Eur Radiol (2020) 30(4):2356–64. doi: 10.1007/s00330-019-06593-y 31900702

[26] Zhao X Chen K Wu G Zhang G Zhou X Lv C. Deep learning shows good reliability for automatic segmentation and volume measurement of brain hemorrhage, intraventricular extension, and peripheral edema. Eur Radiol (2021) 31(7):5012–20. doi: 10.1007/s00330-020-07558-2 33409788

[27] Shelhamer E Long J Darrell T. Fully convolutional networks for semantic segmentation. IEEE T Pattern Anal (2017) 39(4):640–51. doi: 10.1109/TPAMI.2016.2572683 27244717

[28] Liu X Zhang Y Jing H Wang L Zhao S. Ore image segmentation method using U-net and Res_Unet convolutional networks. Rsc Adv (2020) 10(16):9396–406. doi: 10.1039/c9ra05877j PMC9050132 35497237

[29] Bitencourt A Daimiel Naranjo I Lo Gullo R Rossi Saccarelli C Pinker K. AI-Enhanced breast imaging: Where are we and where are we heading? Eur J Radiol (2021) 142:109882. doi: 10.1016/j.ejrad.2021.109882 34392105 PMC8387447

[30] Truhn D Schrading S Haarburger C Schneider H Merhof D Kuhl C. Radiomic versus convolutional neural networks analysis for classification of contrast-enhancing lesions at multiparametric breast MRI. Radiology (2019) 290(2):290–7. doi: 10.1148/radiol.2018181352 30422086

[31] Dalmiş MU Gubern-Mérida A Vreemann S Bult P Karssemeijer N Mann R. Artificial intelligence–based classification of breast lesions imaged with a multiparametric breast MRI protocol with ultrafast DCE-MRI, T2, and DWI. Invest Radiol (2019) 54(6):325–32. doi: 10.1097/RLI.0000000000000544 30652985

[32] Ji Y Li H Edwards AV Papaioannou J Ma W Liu P. Independent validation of machine learning in diagnosing breast cancer on magnetic resonance imaging within a single institution. Cancer Imaging (2019) 19(1):64. doi: 10.1186/s40644-019-0252-2 31533838 PMC6751793

[33] Herent P Schmauch B Jehanno P Dehaene O Saillard C Balleyguier C. Detection and characterization of MRI breast lesions using deep learning. Diagn Interv Imag (2019) 100(4):219–25. doi: 10.1016/j.diii.2019.02.008 30926444

[34] Jiao H Jiang X Pang Z Lin X Huang Y Li L. Deep convolutional neural networks-based automatic breast segmentation and mass detection in DCE-MRI. Comput Math Method M (2020) 2020:1–12. doi: 10.1155/2020/2413706 PMC7232735 32454879

[35] Xu X Fu L Chen Y Larsson R Zhang D Suo S. (2018). Breast region segmentation being convolutional neural network in dynamic contrast enhanced MRI. Annu Int Conf IEEE Eng Med Biol Soc (2018) 2018:750–3. doi: 10.1109/EMBC.2018.8512422 30440504

[36] Chen X Men K Chen B Tang Y Zhang T Wang S. CNN-Based quality assurance for automatic segmentation of breast cancer in radiotherapy. Front Oncol (2020) 10:524. doi: 10.3389/fonc.2020.00524 32426272 PMC7212344

[37] Tsochatzidis L Koutla P Costaridou L Pratikakis I. Integrating segmentation information into CNN for breast cancer diagnosis of mammographic masses. Comput Meth Prog Bio (2021) 200:105913. doi: 10.1016/j.cmpb.2020.105913 33422854

[38] Leithner D Horvat JV Marino MA Bernard-Davila B Jochelson MS Ochoa-Albiztegui RE. Radiomic signatures with contrast-enhanced magnetic resonance imaging for the assessment of breast cancer receptor status and molecular subtypes: initial results. Breast Cancer Res (2019) 21(1):106. doi: 10.1186/s13058-019-1187-z 31514736 PMC6739929

[39] Khaled R Vidal J Vilanova JC Martí R. A U-net ensemble for breast lesion segmentation in DCE MRI. Comput Biol Med (2022) 140:105093. doi: 10.1016/j.compbiomed.2021.105093 34883343

[40] Zhang L Zha Z Qu W Zhao H Yuan J Feng Y. Tumor necrosis as a prognostic variable for the clinical outcome in patients with renal cell carcinoma: a systematic review and meta-analysis. BMC Cancer (2018) 18(1):870. doi: 10.1186/s12885-018-4773-z 30176824 PMC6122538

[41] Fujisaki A Aoki T Kasai T Kinoshita S Tomoda Y Tanaka F. Pleomorphic carcinoma of the lung: Relationship between CT findings and prognosis. Am J Roentgenol (1976) 207(2):289. doi: 10.2214/AJR.15.15542 27144416

[42] Liu Y Wang K Xing H Zhai X Wang L Wang W. Attempt towards a novel classification of triple-negative breast cancer using immunohistochemical markers. Oncol Lett (2016) 12(2):1240–56. doi: 10.3892/ol.2016.4778 PMC4950427 27446423

